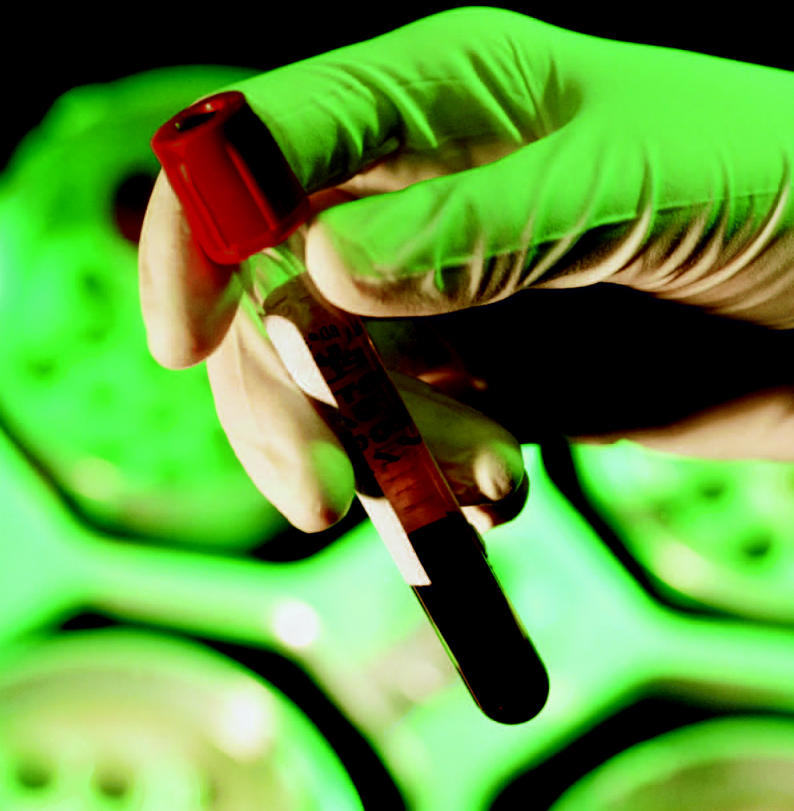# Setting a Baseline for Biomonitoring

**DOI:** 10.1289/ehp.114-a652

**Published:** 2006-11

**Authors:** Harvey Black

Biomonitoring of tissues such as blood, urine, and breast milk is an extremely valuable tool for identifying population exposure to harmful chemicals. The data gathered through biomonitoring can provide guidance on how to prioritize toxicological research, and can result in measures to control and prevent exposure. Despite these benefits, however, “tremendous challenges” still surround the use of this technology, according to *Human Biomonitoring for Environmental Chemicals*, a report released 24 July 2006 by a committee of the National Research Council (NRC). The report noted, for instance, that there should be much more emphasis on communicating the results of studies in the design of the research. It also called for a “consistent rationale for selecting chemicals to be studied based on exposure and public health concerns.”

## More Data than Information

The report was requested in an EPA appropriation in a 2004 House–Senate Conference Report. The NRC committee was charged with the goal of reviewing current practices and recommending ways to improve the interpretation and uses of human biomonitoring data on environmental chemicals.

The committee held four public sessions in which it heard presentations on the conduct and importance of biomonitoring research from a variety of authorities representing government, industry, and academia. Biomonitoring is going to play a major role in the future of environmental health, says committee chair Thomas Burke, a professor of health policy and management at the Johns Hopkins University Bloomberg School of Public Health. “Our biggest challenge is figuring out what this biomonitoring data means in terms of public health, particularly,” he says.

According to the report, biomonitoring has been extremely valuable in tracing time trends in children’s exposure to lead, thus validating the success of public health initiatives such as the removal of lead from gasoline. Biomonitoring has also played an important role in assessing exposures to mercury and secondhand tobacco smoke, and thus in guiding development of exposure prevention strategies.

These success stories notwithstanding, there are few chemicals about which enough is known to make public health pronouncements and provide risk information with confidence. This, the report notes, stands in contrast to scientists’ ability to actually detect chemicals in people. “The problem is that the technology for measurement has in many cases exceeded the available information on animal health effects, let alone human health effects,” says committee member Mark Cullen, a professor of medicine and public health at Yale University School of Medicine.

One reason for the gap is that there is not adequate testing of chemicals in the United States before they are marketed, says Philip Landrigan, a professor of community and preventive medicine and pediatrics at Mount Sinai School of Medicine who peer-reviewed. “We basically assume that chemicals are harmless until some injury is found,” he says.

Carol Henry, a member of the NRC committee and vice president for industry performance programs at the American Chemistry Council (ACC), notes that the difficulty of interpreting biomonitoring data and their relationships to exposure information is a significant barrier to more effective applications of such data—in particular, for implementing intervention strategies.

## Leveraging Resources

“There’s a lot of useful information just about exposure to people that can be gained from doing biomonitoring and not just relying on models and assumptions about what’s getting into people,” says John Balbus, director of health programs at Environmental Defense. The NRC report points to the CDC’s ongoing *National Report on Human Exposure to Environmental Chemicals* as offering the most comprehensive biomonitoring information on a representative sample of the American population. The third *National Report*, issued in July 2005, provides exposure information about 148 chemicals in a sampling of 2,400 people.

Periodic notices in the *Federal Register* ask for chemicals to be nominated for study as part of the *National Report*. A number of criteria are used in selecting the chemicals to be measured by the CDC. These include the degree to which people are exposed to them, the gravity of the known or suspected health effects, the availability of accurate, repeatable methods to measure the chemicals, and the cost of testing. Scientists at the CDC’s National Center for Environmental Health (NCEH) and outside reviewers use these criteria to make the final selection.

The CDC report is not a report on health, asserts John Osterloh, chief medical officer at the NCEH Division of Laboratory Sciences; it is a report on exposure. “We’re very cautious in the report to say that we don’t know whether or not the levels are related to health effects,” he says. “Better exposure information will produce better decisions to protect people’s health.” The exposure information, he says, can steer science toward studying possible health effects of chemicals that are in people.

The NRC report points to changes it would like to see in the way the CDC goes about this survey. “We want to improve the chemical selection process to include more input,” says Burke. “We feel there should be a multiagency group to look at our existing body of knowledge to make sure we’re looking at the right kinds of things. We would like to get the program to look at what emerging issues might be, based upon both potential for population exposure and evidence of potential public health impacts.” The report notes that the agencies routinely involved in selecting chemicals should include not just the CDC but also the EPA, the NIEHS, the National Toxicology Program, the FDA, and the USDA.

The NRC committee further states that the CDC should improve its efforts to communicate the results of its survey to the public, agency members, and policy makers. “The CDC comes out with a report on exposure, but really leaves it up to the media to interpret the potential implications,” says Burke. According to the NRC report, communicating results of biomonitoring has not played as important a role as it should—too often in such studies, interpreting and communicating the results would appear to have been “an afterthought,” notes Burke.

The design of communication strategies should take a higher priority so the public can be better informed. The strategy should include being ready to answer important questions that the public will ask, Burke says, such as why the chemical was selected and what is and isn’t known about it. “This [strategy] can be designed up front to facilitate the process,” he says.

When there aren’t answers to such questions, Burke says, it’s perfectly legitimate for scientists to voice their ignorance. “But,” he adds, “when you don’t know, it’s really important to communicate how you’re going about learning.”

But how does one craft messages about the presence of environmental chemicals in people’s bodies? As the report notes, “Absence of evidence of effects is not identical with evidence of absence of effects—a distinction that must be made clear to constituents.” Researchers may sometimes appropriately conclude that, while high biomarker levels are not necessarily bad, low levels are not necessarily good; sometimes it is difficult to come up with easy answers to questions on the health impact of chemicals. The NRC committee calls for empirical research on how to convey such a conclusion without engendering the twin problems of baseless concern or apathy.

## Closing the Gaps

The NRC report also points to the need for research that applies biomarkers for environmental chemicals in animal toxicology testing to understand how these biomarkers relate to adverse effects. “Right now we know what [test animals’] intake dose is, but we don’t know how that translates into biomarker levels,” says Burke. “For example, it would be so much more informative to the interpretation process if we could compare blood levels in test animals to blood levels in humans.” Traditionally, he explains, toxicology studies have not examined blood levels in animal models.

As one example he points to perfluorooctanoic acid (PFOA), which is used in making Teflon and materials used in many consumer products such as food packaging and stain-resistant clothing. “Although we have found that there is widespread presence [of PFOA] in the environment and people, we have limited testing data to help us understand just how much the low levels found throughout the population might impact public health,” says Burke. (Despite the uncertainty about the health effects of these low levels of PFOA, DuPont and other chemical companies using the compound pledged in January 2006 to work toward eliminating it by 2015.)

The need to make sense of human biomonitoring data and understand them as markers of exposure stimulated the ACC to hold a workshop shortly after the NRC report was issued, with the goal of identifying knowledge gaps and research needs. Among the concerns examined is a critical need for understanding what is found in the body and where and how the exposure occurred, explains Tina Bahadori, a senior scientist with the ACC who co-chaired the workshop. “Ultimately the goal is to know how you would intervene to either modulate the exposure or ascertain for sure there is no concern. If you don’t know where and how it came from, there is no way you can do that,” she says.

Biomonitoring has also drawn the attention of the California legislature. Law makers this year have passed a bill to set up a state biomonitoring program, which would sample state residents on a voluntary basis. According to Richard Jackson, an adjunct professor of environmental health sciences at the University of California, Berkeley, such a program is important, considering some of the characteristics of chemical use in the state. “California uses well over a quarter of the most toxic pesticides in the nation,” Jackson says. “We have a series of high-tech industries in the state that use chemicals that are really not used in other parts of the country.” As former director of the NCEH, Jackson led the CDC’s biomonitoring program from 1994 to 2003.

The new law makes California the first state to legislate a biomonitoring program. A 16-member scientific advisory panel will be appointed by legislative leaders and the heads of the California EPA and Department of Health Services by 1 July 2007 to recommend design and implementation of the program. The program would select communities that would be “reflective of the economic, racial, and ethnic composition of the state,” according to the law. The law, however, also says that biomonitoring samples may be taken from so-called nongeographical communities—that is, people who may share a common chemical exposure because they have similar jobs or lifestyles.

As the California legislation and the NRC report demonstrate, biomonitoring is becoming an important tool in assessing environmental health. The report describes it as “a potentially powerful new lens for examining public exposure to toxic chemicals.” Yet as the report also makes plain, effective use of this tool demands that the research community address a number of technical and communication challenges.

## Figures and Tables

**Figure:**